# Predicting Fazekas scores from automatic segmentations of white matter signal abnormalities

**DOI:** 10.18632/aging.102662

**Published:** 2020-01-12

**Authors:** Nira Cedres, Daniel Ferreira, Alejandra Machado, Sara Shams, Simona Sacuiu, Margda Waern, Lars-Olof Wahlund, Anna Zettergren, Silke Kern, Ingmar Skoog, Eric Westman

**Affiliations:** 1 Division of Clinical Geriatrics, Centre for Alzheimer Research, Department of Neurobiology, Care Sciences, and Society, Karolinska Institutet (KI), Stockholm, Sweden; 2 Department of Clinical Neuroscience, KI, Stockholm, Sweden; 3 Department of Radiology, Karolinska University Hospital, Stockholm, Sweden; 4 Centre for Ageing and Health at The University of Gothenburg, Gothenburg, Sweden; 5 Neuropsychiatric Epidemiology Unit, Department of Psychiatry and Neurochemistry, Institute of Neuroscience and Physiology, Sahlgrenska Academy at The University of Gothenburg, Gothenburg, Sweden; 6 Department of Neuroimaging, Centre for Neuroimaging Sciences, Institute of Psychiatry, Psychology and Neuroscience, King’s College London, London, UK; 7 Region Västra Götaland, Sahlgrenska University Hospital, Department of Neuropsychiatry, Gothenburg, Sweden; 8 Region Västra Götaland, Sahlgrenska University Hospital, Psychosis Department, Gothenburg, Sweden

**Keywords:** white matter, visual rating, hyperintensities, hypointensities, Fazekas scale

## Abstract

Different measurements of white matter signal abnormalities (WMSA) are often used across studies, which hinders combination of WMSA data from different cohorts. We investigated associations between three commonly used measurements of WMSA, aiming to further understand the association between them and their potential interchangeability: the Fazekas scale, the lesion segmentation tool (LST), and FreeSurfer. We also aimed at proposing cut-off values for estimating low and high Fazekas scale WMSA burden from LST and FreeSurfer WMSA, to facilitate clinical use and interpretation of LST and FreeSurfer WMSA data. A population-based cohort of 709 individuals (all of them 70 years old, 52% female) was investigated. We found a strong association between LST and FreeSurfer WMSA, and an association of Fazekas scores with both LST and FreeSurfer WMSA. The proposed cut-off values were 0.00496 for LST and 0.00321 for FreeSurfer (Total Intracranial volumes (TIV)-corrected values). This study provides data on the association between Fazekas scores, hyperintense WMSA, and hypointense WMSA in a large population-based cohort. The proposed cut-off values for translating LST and FreeSurfer WMSA estimations to low and high Fazekas scale WMSA burden may facilitate the combination of WMSA measurements from different cohorts that used either a FLAIR or a T1-weigthed sequence.

## INTRODUCTION

The Fazekas scale [1] is a widely used method to visually rate hyperintense white matter signal abnormalities (WMSA) in magnetic resonance imaging (MRI) data, both in clinical practice and research [1–4]. High Fazekas WMSA burden (i.e. confluent WMSA, Fazekas 2 or 3 scores) is associated with greater cognitive dysfunction than low Fazekas WMSA burden (i.e. absence or punctate WMSA, Fazekas 0 or 1 scores) in population-based cohorts [5]. Concordantly, in clinical settings high Fazekas WMSA burden has successfully predicted cognitive performance in Alzheimer’s disease patients [6]. High cerebrovascular pathology burden has clinical relevance for diagnosis of individuals with higher risk for cerebrovascular disease [4]. Despite great utility, visual ratings of WMSA are subjective, which often compromises inter-rater reliability. Automated methods have emerged as objective alternatives. The Lesion Segmentation Tool (LST) (https://www.applied-statistics.de/lst.html) [7] is widely used to automatically segment WMSA in the form of hyperintensities in the T2-weighted fluid-attenuated inversion recovery (FLAIR) MRI sequence. Previous studies have shown strong correlations between the Fazekas scale and automatic segmentations of hyperintense WMSA [2, 3]. Also, the FreeSurfer software (https://surfer.nmr.mgh.harvard.edu/) [8] is increasingly used to automatically segment WMSA in the form of hypointensities in the T1-weighted MRI sequence [9–13]. To our knowledge, the association between hypointense WMSA and the Fazekas scale has not been investigated yet. Furthermore, a limitation of automated methods is that they are complex, time consuming, require quality control, and lack normative data, all of which compromise their clinical use at present.

Regarding the association between automatic segmentations of hyperintense and hypointense WMSA, studies are scarce but some data show a close correlation between the two [2, 14]. However, the underlying pathology reflected by hyperintense and hypointense WMSA is not yet entirely understood and they may reflect different microstructural tissue properties. Hypointense WMSA seems to be related to poorer white matter integrity as compared to hyperintense WMSA [14]. On the other hand, hyperintense WMSA may reflect a mix of white matter damage, peri-inflammatory processes, and other pathologies related to increased cerebrovascular and blood-brain barrier permeability [15].

Understanding how these three measurements relate to each other, endorsing their potential interchangeability, is of utmost importance because WMSA are frequently assessed with different methods and MRI sequences across studies. This makes it difficult to combine data from different cohorts. Hence, our primary aim was to investigate the association between the Fazekas scale, hyperintense WMSA based on the LST software, and hypointense WMSA based on the FreeSurfer software, in order to understand their association and potential interchangeability, in a large population-based cohort. Our secondary aim was to propose reliable cut-off values for automatic segmentations of WMSA data from LST and FreeSurfer to identify low and high Fazekas scale WMSA burden. These cut-off values might potentially facilitate combination of WMSA data from different cohorts, software, and MRI sequence types (FLAIR and T1-weigthed), as well as facilitate clinical use and interpretation of LST and FreeSurfer WMSA data.

## RESULTS

**Figure 1:**
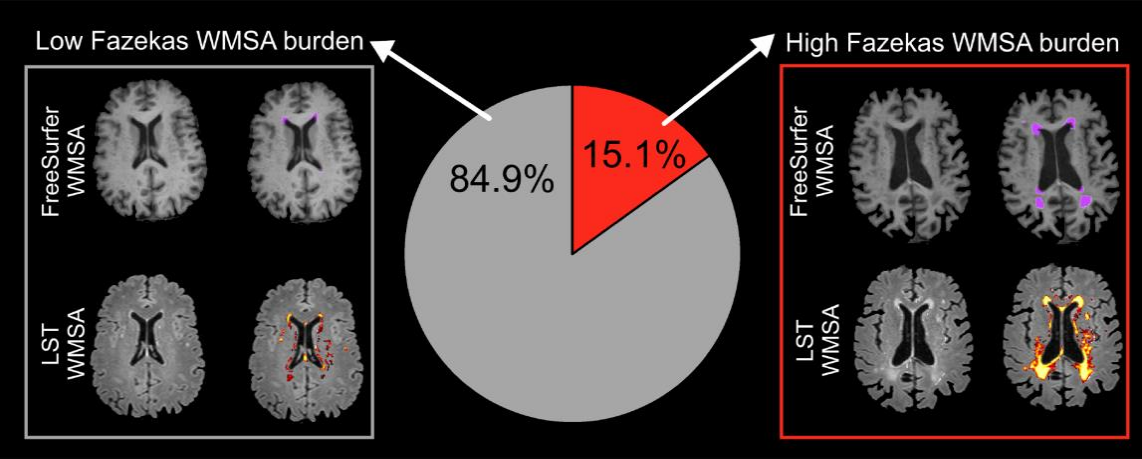
**Prevalence of low and high Fazekas WMSA burden.** Low WMSA burden was defined as Fazekas scores 0 (i.e. absence of WMSA) or 1 (i.e. punctate WMSA). High WMSA burden was defined as Fazekas scores 2 (i.e. early confluent WMSA) and 3 (i.e. WMSA in large confluent areas). The gray box illustrates automatic segmentations of WMSA by FreeSurfer (first row) and LST (second row), for a representative subject with low Fazekas WMSA burden. The red box illustrates automatic segmentations of WMSA by FreeSurfer (first row) and LST (second row), for a representative subject with high Fazekas WMSA burden. WMSA: White matter signal abnormalities; LST: Lesion segmentation tool.

In our population-based cohort of 709 individuals (all 70 years old, 52% female), 15.1% had high Fazekas WMSA burden (i.e. scores 2 and 3) and the rest had low WMSA burden (i.e. Fazekas scores 0 and 1) (Figure 1). After adjusting WMSA volume in milliliters by each participant total intracranial volume (TIV), the mean LST WMSA volume was 0.0043 (SD=0.0054) and the mean FreeSurfer WMSA volume 0.0028 (SD=0.0033) (mean uncorrected WMSA volumes were 6.6 ml (SD = 8.6) for LST and 4.3 ml (SD = 5.3) for FreeSurfer).

**Figure 2:**
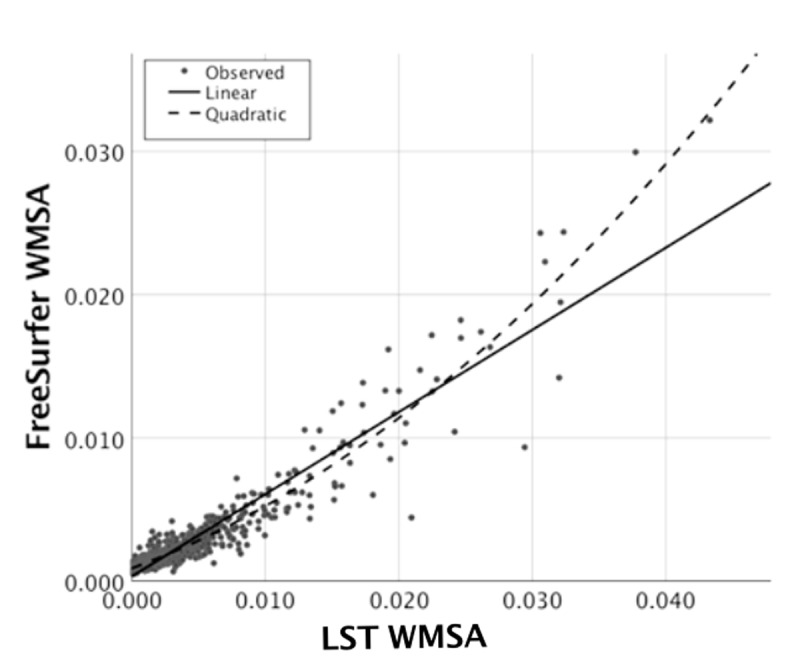
**Association between hyperintense WMSA based on the LST software and hypointense WMSA based on the FreeSurfer software.** The Figure shows the linear and quadratic association between LST WMSA (x axis) and FreeSurfer WMSA (y axis) volume in milliliters after adjusting for each participant’s TIV. WMSA: White matter signal abnormalities; TIV: total intracranial volume; LST: Lesion segmentation tool.

**Figure 3:**
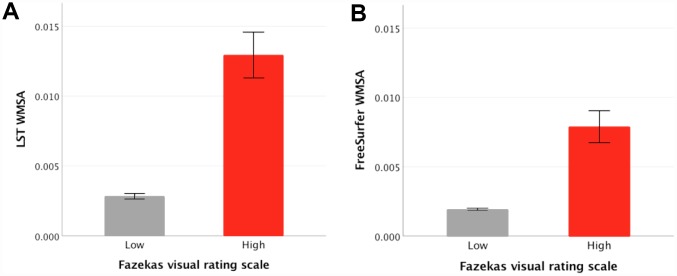
**Mean differences between low and high Fazekas WMSA burden in hyperintense WMSA from LST and hypointense WMSA from FreeSurfer.** (**A**) shows FreeSurfer WMSA levels for low and high Fazekas scores, error bars represent the standard error; (**B**) shows LST WMSA levels for low and high Fazekas scores, error bars represent the standard error; The y axis represents WMSA volumes in milliliters after adjusting for each participant’s TIV. WMSA: White matter signal abnormalities; LST: Lesion segmentation tool; TIV: total intracranial volume.

Regarding our primary aim, the association between LST and FreeSurfer WMSA estimations is displayed in Figure 2. There was a strong linear association between LST and FreeSurfer WMSA (Rxy=0.939; p<.001). The quadratic association between LST and FreeSurfer WMSA was also significant (β_1_=0.563; p<.001; β_2_=0.413; p<.001). The paired-sample t-test revealed that LST WMSA volumes were significantly larger than FreeSurfer WMSA volumes (t_(708)_=-15.9;p<.001). Visual inspection of Figure 2 suggests that this difference between LST and FreeSurfer WMSA volumes is more prominent in the range of small WMSA. The association between Fazekas and hyperintense WMSA (t_(109.1)_ = −12.1;p<.001) and between Fazekas and hypointense WMSA (t_(107.1)_=-10.1;p<.001) were also significant, revealing larger volumes for the high Fazekas scale WMSA burden category (Figures 3A and 3B).

**Figure 4:**
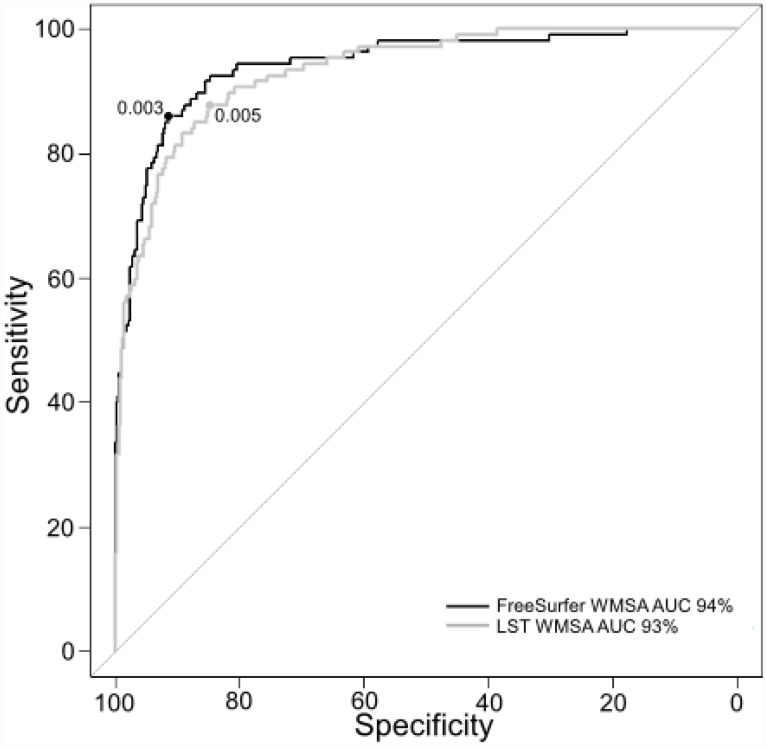
**ROC curves for separating low and high Fazekas WMSA burden.** The figure shows the ROC curves for separating low and high Fazekas WMSA burden for LST and FreeSurfer WMSA values (AUC and optimal cut-off values are shown for each software type). Fazekas scores were categorized as low WMSA burden (scores = 0 and 1) or high WMSA burden (scores = 2 and 3). WMSA: White matter signal abnormalities; AUC: Area under the curve; ROC: Receiver operating characteristic; LST: Lesion segmentation tool.

Regarding our secondary aim, we performed a receiver operating characteristic (ROC) curve in order to obtain reliable cut-off values for automatic segmentations of WMSA data from LST and FreeSurfer and identify low and high Fazekas scale WMSA burden. The ROC analyses separating low and high Fazekas WMSA burden categories revealed an AUC value of 93% for LST WMSA, and an AUC value of 94% for FreeSurfer WMSA (Figure 4). The proposed cut-off value to classify low and high Fazekas WMSA burden is 0.00496 for LST WMSA, and 0.00321 for FreeSurfer WMSA. These values are adjusted by the TIV. Corresponding sensitivity and specificity values were 88% and 85% for LST WMSA, and 86% and 91% for FreeSurfer WMSA.

## DISCUSSION

In a large population-based cohort of 70-year old individuals, 15.1% had a high Fazekas WMSA burden. This frequency is, as expected, higher than that reported in younger population-based cohorts (frequency of 10%) [16] and lower than that reported in older cohorts (frequencies between 26–36%) [3, 17]. Our correlation analyses showed a strong association between LST and FreeSurfer WMSA. In line with previous studies [2, 14], we also found that LST WMSA volumes were larger than FreeSurfer WMSA volumes, especially in the range of small WMSA. A possible explanation for this finding is that hyperintense and hypointense WMSA may reflect different microstructural tissue properties. Hypointense WMSA are related to poorer white matter integrity as compared to hyperintense WMSA [14], suggesting that hypointense WMSA are more closely related to necrotic damage. In contrast, more acute white matter damage from different etiologies may be captured by hyperintense WMSA, leading to larger WMSA volumes [2, 14], covering peri-inflammatory processes and other pathologies related to increased cerebrovascular burden and blood-brain barrier permeability [15]. The association between Fazekas and hyperintense WMSA, and between Fazekas and hypointense WMSA was also significant. Larger volumes were observed for the high Fazekas scale WMSA burden category. These results are in line with previous studies [2, 3, 14]. Nevertheless, to our knowledge, our study is the first reporting associations between Fazekas scores, hypointense WMSA, and hyperintense WMSA in the same cohort.

Regarding the ROC analyses, both automatic segmentation methods could separate low and high Fazekas scores with high sensitivity and specificity. Since the proposed cut-offs values for low and high Fazekas WMSA burden are adjusted for the TIV, we believe that these values may be more easily transferred to volume estimations from other MRI scanners, when similar MRI scanning protocols are used. There may still be some source of error related to inter-rater variability for the Fazekas scale. However, inter- and intra-rater reliability is usually higher for the Fazekas scale than for other visual rating scales [18].

Some limitations should be mentioned. Despite that Fazekas visual rating does not seem to be influenced by the experience of the rater [19], future studies should confirm that the experience of the rater does not influence the cut-offs for predicting Fazekas scores from LST and FreeSurfer”. Regarding MRI acquisition, although our two sequences were acquired in the same scanner, the slice thickness was thicker for the FLAIR sequence than for the T1-weigthed sequence (2.0 mm and 1.0 mm respectively), which may influence volumetric results. However, given the quadratic association between both automated measures and previous findings showing similar results [2, 14, 15], we suggest that our findings are related to different underlying pathology rather than the differences in their slice thickness. The association of Fazekas scores, LST WMSA, and FreeSurfer WMSA with demographic, cognitive, and clinical factors has been investigated in previous studies [4, 5, 16, 17, 23, 24] and is beyond the scope of our study. We decided to focus on the association between these three methods and their potential interchangeability, as well as on deriving cut-off values for predicting high and low Fazekas scores from automatic segmentations of WMSA. Future studies should test and validate our findings in other population-based cohorts and in clinical settings, testing the clinical applicability of our cut-off values: e.g. to discriminate between clinical entities (diagnostic capacity) and predict future cognitive decline (prognostic capacity). Further, our findings should be tested in studies including different raters (ideally with different levels of expertise), and different MRI scanners.

In conclusion, we provide data on the association between Fazekas scores, hyperintense WMSA, and hypointense WMSA in a large population-based cohort. Validating our proposed cut-off values in other cohorts is of great importance for future studies combining WMSA measurements based on different MRI sequences (FLAIR and T1-weigthed).

## MATERIALS AND METHODS

### Participants

As part of the Gothenburg H70 Birth Cohort Studies all men and women born 1944 on dates ending with 0, 2, 5, or 8, and registered as residents in Gothenburg according the Swedish Tax Agency were invited to a comprehensive examination on ageing and age-related factors [25]. Individuals were invited irrespective of their place of living (e.g. private households, sheltered living). A total of 1203 (response rate 72.2%; 559 men and 644 women; mean age 70.5 years) agreed to participate. The general examinations and various procedures have been described in detail previously [25]. All study participants were invited to take part in a brain imaging examination, conducted at the Aleris Clinic in Gothenburg. An MRI examination was conducted in 792 individuals (response rate 65.8%). Global cognitive status as measured by the Mini-Mental State Examination (MMSE) ranged between 0 and 30 (mean = 28.8, SD = 2.4). 97.7% of participants were cognitively normal or had mild cognitive impairment (no dementia). Clinical Dementia Rating (CDR) distribution was CDR 0 = 71.3%; CDR 0.5 = 24.9%; and CDR 1 ≥ 3.9%.

For the current study, inclusion criteria were: (1) availability of Fazekas visual ratings; and (2) outcomes for both LST and FreeSurfer suitable for analysis (e.g. no processing errors, etc.), giving a sample of 709 individuals (52% female). MMSE ranged between 20 and 30 (mean = 29, SD = 1). The majority of participants (99.3%) were cognitively normal or had mild cognitive impairment (no dementia). CDR distribution was CDR 0= 81.8%; CDR 0.5= 18.1%; and CDR 1= 0.1%.

### MRI data acquisition, image processing, and measurements of WMSA

MRI data were acquired in a 3.0T Philips Achieva system (Philips Medical Systems), using a 3D T1-weigthed Turbo Field Echo (TFE) sequence (RT=7.2 ms., ET=3.2 ms., flip angle=9°, number of slices=160, matrix size=250x250 mm, slice thickness=1.0 mm); and a 3D FLAIR sequence (RT=48000 ms., ET=280 ms., TI=1650 ms., flip angle=90°, number of slices=140, matrix size=250x237 mm, slice thickness=2.0 mm).

The Fazekas scale was applied on FLAIR MRI data on the axial plane and was scored following standard guidelines [1]. Briefly, Fazekas grades WMSA as 0 (i.e. absence of WMSA), 1 (i.e. punctate WMSA), 2 (i.e. early confluent WMSA), and 3 (i.e. WMSA in large confluent areas). The frequently used Fazekas clinical classification in low and high WMSA burden [16, 24, 26] was applied to address the secondary aim of this study. High WMSA burden was defined as Fazekas scores 2 or 3. Low WMSA burden was defined as Fazekas scores 0 or 1. Fazekas ratings were done by an experienced neuroradiologist (S.S.), who has participated as a rater in several previously published studies with excellent inter-rater agreement (weighted k and intra-class correlation coefficient >0.90) [6, 21, 27, 28].

WMSA were also automatically segmented with LST 2.0.15 and FreeSurfer 6.0.0. LST is an open source segmentation toolbox implemented in the SPM software (https://www.fil.ion.ucl.ac.uk/spm/). LST utilizes a lesion prediction algorithm (LPA) based on FLAIR images intensity distribution (hyperintensities) that builds a lesion probability map for each individual. The T1-weighted images were processed with the FreeSurfer image analysis suite. FreeSurfer detects hypointensities and automatically labels WMSA volumes for each participant using a probabilistic procedure as well [8]. MRI data management and processing was done with our database system theHiveDB [29]. WMSA volumes in milliliters (ml) from both LST and FreeSurfer were adjusted by the TIV, estimated with SPM12, by dividing each participant’s WMSA volume by their corresponding TIV [30].

### Statistical analysis

Associations between LST and FreeSurfer WMSA estimations were tested using the Pearson correlation. Quadratic simple regression was also used to test for the association between LST and FreeSurfer WMSA. Paired-sample t-test was used for the comparison of LST and FreeSurfer WMSA mean volumes. Two independent-sample t-tests were used for the comparison of low (scores = 0 and 1) and high (scores = 2 and 3) Fazekas scale WMSA burden categories in LST and WMSA mean volumes. The cut-off value for estimating low and high Fazekas scale WMSA burden from LST and FreeSurfer WMSA was derived as follows. First, Fazekas scores were categorized as low WMSA burden or high WMSA burden [1, 26]. Second, the area under the receiver operating characteristic (ROC) curve was calculated to identify the LST and FreeSurfer WMSA values that best separated low and high Fazekas scale WMSA burden categories. Third, sensitivity and specificity values based on the ROC curves were calculated to provide interpretable parameters of the classification. Statistical analyses were conducted using the R statistical software (http://www.r-project.org). A p-value <0.05 (two-tailed) was deemed significant in all the analyses.

### Ethical approval

The H70 study was approved by the Regional Ethical Review Board in Gothenburg (Approval Numbers: 869-13, T076- 14, T166-14, 976-13, 127-14, T936-15, 006-14, T703-14, 006-14, T201-17, T915-14, 959-15, T139-15), and by the Radiation Protection Committee (Approval Number: 13-64) in concordance with the 1964 Helsinki declaration and its later amendment.
